# Transition to Phacoemulsification at the Farabi Eye Hospital, Iran

**DOI:** 10.4103/0974-9233.80709

**Published:** 2011

**Authors:** Hassan Hashemi, S-Farzad Mohammadi, Arash Mazouri, Mercede Majdi-N, Mahmoud Jabbarvand, Hadi Z-Mehrjardi

**Affiliations:** 1 Eye Research Center, Farabi Eye Hospital, Tehran University of Medical Sciences, Tehran, Iran; 2 Noor Ophthalmology Research Center, Noor Eye Hospital, Tehran, Iran; 3 Students’ Scientific Research Center, Tehran University of Medical Sciences, Tehran, Iran

**Keywords:** Cataract Surgery, Knowledge Transfer and Exchange, Phacoemulsification

## Abstract

**Purpose:**

To provide objective evidence on the transition of cataract surgical care at Farabi Eye Hospital, Iran.

**Materials and Methods:**

Two separate years, 2003 and 2006, were selected for evaluation. One thousand nine hundred fifty-seven surgical records of age-related cataract cases were randomly selected and reviewed. Three hundred fifty-three patients (405 eyes) in 2006 and 125 patients (153 eyes) in 2003 were selected randomly for a follow-up examination. The two phases were compared in terms of surgical routines, patient characteristics and outcomes for statistical differences. *P* <0.05 was considered statistically significant.

**Results:**

The phacoemulsification rate increased from 25% to greater than 90% between 2003 and 2006, rates of corneal incisions and use of foldable intraocular lenses tripled, administration of general anesthesia dropped from 80% to 12%, the outpatient admission rate rose from 5.2% to 71%, 4% vs. 66% of the operations were performed by a senior phacoemulsification surgeon and the number of advanced surgeons changed from 6% to 38% (all *P*-values < 0.001). In 2006, more patients at the two extremes of age, more patients with poor systemic conditions and myopes underwent surgery (all *P*-values < 0.05); the cataract surgery volume increased by 49% and post-operative visual acuity improved (*P* = 0.03) while patient satisfaction was unchanged.

**Conclusion:**

We objectively documented the transition in cataract surgery technique to phacoemulsification at the Farabi Eye Hospital in the mid-2000s. This was accompanied by significant expansion of the spectrum of cataract surgery candidates and remarkable attainment of surgical skill.

## INTRODUCTION

Vision 2020: the Right to Sight, a joint initiative of the World Health Organization (WHO) and the International Agency for the Prevention of Blindness (IAPB) aims to eliminate avoidable blindness in the world by the year 2020.^1^ It is estimated that over 90% of the world’s visually impaired live in developing countries, where blindness is associated with considerable disability, resulting in major economic and social consequences.^2^,^3^ Vision 2020 is based on disease control and infrastructure and human resource development, but it does not explicitly include a comprehensive plan for knowledge and technology transfer and exchange, which is relevant to eye care in developing countries.^4^

Cataract is the leading cause of treatable blindness in the world.^5^ Advances in the science of and techniques for cataract extraction make this procedure one of the most successful and common surgical procedures in medicine. Cataract surgery has evolved dramatically in the recent years in Iran.^6^ Even a decade ago, some senior ophthalmologists in the capital, Tehran, occasionally performed intracapsular cataract extraction (ICCE). Phacoemulsification is now considered the state-of-the-art approach for cataract extraction in the world, but introduction of phacoemulsification in developing countries remains hampered.

The aim of this study was to provide tangible evidence on the changes in the procedures for cataract care – admission, surgical technique, anesthesia, etc. – in the largest eye referral center in Iran. We describe the skill acquisition profile in the time frame of the study and assess the impact of such knowledge and technology transfer in service delivery in terms of patient characteristics and outcomes.

## MATERIALS AND METHODS

**Figure 1:**
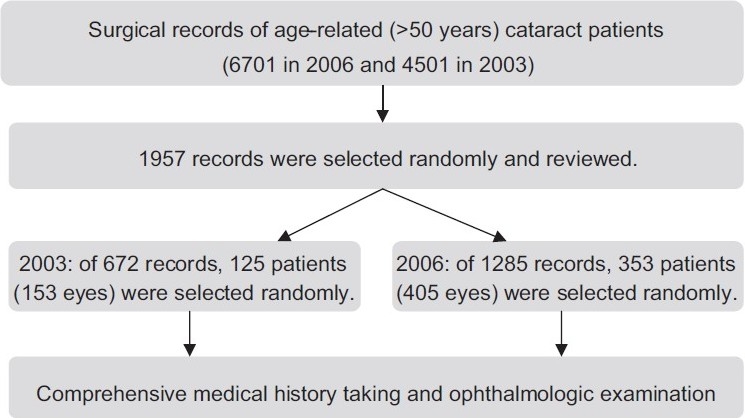
Participant flow in the cataract surgery outcome study of Farabi Eye Hospital, Tehran, Iran

This study was conducted at the Farabi Eye Hospital, which is the largest university-affiliated eye referral center in Iran with 450 beds. Farabi Eye Hospital has a current annual volume of about 13,300 cataract surgeries (about 40% of the hospital’s total annual surgical procedures) and more than 300,000 visits. Two periods, January 2003 to January 2004 (2003 group) and January 2006 to January 2007 (2006 group), were chosen. A total of 1957 related charts were reviewed: 1285 ocular surgery charts of patients aged over 50 years and 672 ocular surgery charts of others, selected based on simple randomization from 6701 and 4501 hospital records from 2006 and 2003, respectively. Data were collected from the patient charts by a trained team, and 353 cataract patients (405 eyes) in 2006 and 125 patients (153 eyes) in 2003 were selected randomly and requested to attend a follow-up examination. Figure 1 shows the flow of participants in the study. The comprehensive study protocol will be reported elsewhere.

Post-operative uncorrected visual acuity (UCVA) and satisfaction were considered as the outcome variables. The VA was measured using chart projectors (CP – 670 20/10–20/400, Nidek Co., Gamagori, Japan) and E letters at a distance of 4 m. The monocular VA was recorded as the smallest line at which the patient could read four letters correctly. If a person was unable to read the largest E (20/400) on the chart at 4 m, the vision was recorded as count fingers, hand motions and light perception based on subject response. Subjects were verbally queried regarding satisfaction with the surgical outcome and were categorized into two groups based on the response: “satisfied” or “dissatisfied.” In subjects who underwent bilateral cataract surgery, satisfaction for each eye was recorded separately.

The two phases were compared in terms of surgical routines, patient characteristics and patient outcomes. The framework included 1957 eyes of cataract patients, which was the denominator for all of the variables, except the factors that were merely evaluable in the follow-up exam, including wound characteristics and outcomes variables for which the denominator was 558. The Chi-square test was used for comparison when appropriate. To compare the mean and distribution of age between groups, the independent samples *t* test and Levene’s test for equality of variances were used, respectively. Although UCVA data was converted to a logMAR scale for the analyses, it did not match the normal distribution, and non-parametric test (Mann–Whitney) was used to compare the visual outcome between the two phases. *P* <0.05 was considered statistically significant.

Surgeon competence was assessed based on qualification and experience. A junior resident was considered a novice surgeon; a non-cornea specialized faculty member, a senior resident, a non-cornea specialized fellow and a non-academic general ophthalmic surgeon were considered intermediate surgeons; and a cornea fellow and the institution’s cataract surgeons with the most years of practice were considered advanced phaco surgeons. The suitability of ranks allocation and skill attainment was reviewed by the head of the Cornea Division [HH] of the Hospital.

We defined myopic subjects as the cases who had received a lens of less than 19 D.

To increase the validity of measuring the rate of intraocular lens (IOL) injector use, cases of extracapsular cataract extraction in which the injector use was not applicable were excluded from the denominator of the rate.

The integrity and ethical aspects of the study protocol were approved by Tehran University of Medical Sciences Research Council.

## RESULTS

Subjects who were examined in the study were comparable to the population framework in terms of sex (female participants vs. non-participants: 49% vs. 53%), mean age at the time of surgery (67 years vs. 69 years) and intraocular lens (IOL) power (19.9 D vs. 20 D).

Mean age of the subjects at the time of surgery was comparable in both groups (range, 50–93 years, mean 68.5 in 2003; and range 50–104 years, mean 68.5 in 2006; *P* = 0.99). Subject age was statistically more evenly distributed in the 2006 group compared with the 2003 group (SD = 9.7 in 2006 vs. SD = 9.1 in 2003; *P* = 0.02). More patients at the two extremes of age (more than 80 years and less than 60 years) were offered cataract extraction: 28% in 2003 vs. 32.3% in 2006 (*P* = 0.05). The sex distribution was equally distributed in both groups (*P* = 0.28) [Table 1].

**Table 1:** Changing trends in demographics of cataract patients at the Farabi Eye Hospital, Iran: 2003 vs. 2006 (population framework)

Patient characteristics	2003	2006	*P*-value*
Age (years)			
Mean	68.5	68.5	0.99
SD	9.1	9.7	0.02
<60 or >80 (%)	28.0	32.3	0.05
Sex (% female)	50.7	52.4	0.28
Systemic conditions (%)			
DM	20.3	22.2	0.72
Others: HTN, HLP or IHD	37.3	49.4	0.01
Myopic patients (%)	13.9	19.3	<0.01

SD: Standard deviation, DM: Diabetes mellitus; HTN: Hypertension, HLP: Hyperlipidemia; IHD: Ischemic heart disease,

* Independent samples t test and Levene’s test for equality of variances were used to compare the mean and SD of age between the two phases. For other variables, Chi-square significance is presented. *P*<0.05 is statistically significant

Cataract surgery was provided statistically more frequently for subjects with systemic conditions in 2006 (*P* = 0.01) [Table 1]. However, the rate of surgery in diabetics did not change significantly from 2003 to 2006 (*P* = 0.72) [Table 1].

Between 2003 and 2006, there was a statistically significant increase of 65.8% in phacoemulsification rate (P < 0.001) [Table 2]. At the same time, there was more than a 20% drop in the rate of small-incision cataract surgery (SICS) and 40% drop in the rate of extracapsular cataract extraction (ECCE) [Table 2].

**Table 2:** Cataract surgery at Farabi Eye Hospital, Iran: 2003 vs. 2006

Parameter	2003	2006	*P*-value*
Surgical routines (%)			
Surgical technique			<0.001
Phacoemulsification	166 (25)	1160 (90.8)	
SICS	188 (28.2)	57 (4.5)	
ECCE (and “converted”)	311 (46.8)	60 (4.7)	
Temporal approach	45 (29.4)	362 (89.4)	<0.001
Corneal incision	38 (24.8)	302 (74.6)	<0.001
Injector use (in case of phacoemulsification)	10 (22.7)	92 (24.7)	0.41
IOL type			
Foldable	52 (34.9)	390 (99.5)	<0.001
Hydrophilic	48 (68.6)	287 (86.4)	0.001
Anesthesia (local)	29 (19.2)	355 (87.4)	<0.001
Admission type (outpatient)	8 (5.2)	287 (70.9)	<0.001
Skill attainment (%)			
Surgeons’ skill level in operations			<0.001
Low	137 (89.5)	51 (12.6)	
Moderate	10 (6.5)	85 (21)	
High	6 (3.9)	269 (66.4)	
Phaco surgery skill among surgeons			<0.001
Low	87	21	
Moderate	7	41	
High	6	38	

SICS: Small incision cataract surgery, ECCE: Extracapsular cataract extraction, IOL: Intraocular lens,

* Chi-square significance was used, *P*<0.05 is statistically significant

During the transition, the number of procedures using the corneal incision technique tripled and the number of operations using the limbal and scleral incision techniques decreased (*P* < 0.001) [Table 2]. There was a statistically significant increase in implantation of hydrophilic IOLs (*P* < 0.001) [Table 2]. Implantation of foldable IOL almost tripled from 2003 to 2006, with almost all subjects receiving foldable lenses (*P* < 0.001) [Table 2]. In 2006, less than 12% of the cataract surgery was performed under general anesthesia, whereas more than 80% was performed under similar conditions in 2003 (*P* < 0.001), and the use of topical anesthesia was just emerging (none in 2003 and 1.2% in 2006). The rate of hospitalization was statistically lower in 2006 (*P* < 0.001).

In 2003, only 3.9% of the patients underwent surgery by a senior phacoemulsification surgeon, compared with 66.4% in 2006 (*P* < 0.001). Statistically fewer surgical procedures were performed by novice ophthalmologists in 2006 than in 2003 (*P* < 0.001) [Table 2].

Subjective satisfaction was statistically similar between the groups (*P* = 0.57). On the other hand, the median post-operative UCVA improved significantly from 0.26 to 0.22 LogMAR (0.55-0.6 based on decimal acuity scale, respectively; *P* = 0.03). During the transition between 2003 and 2006, cataract surgery volume increased by 49% at the hospital [Table 3]. Between 2003 and 2006, the ratio of annual volume of cataract surgeries to the hospital’s total annual surgical procedures increased by 15%.

**Table 3:** Changing trend in outcomes in cataract patients at Farabi Eye Hospital, Iran: 2003 vs. 2006

Outcomes	2003	2006	*P*-value^*^
Satisfied (%)	75.8	79.5	0.57
UCVA (LogMAR)	0.26	0.22	0.03
Hospital annual cataract surgery volume	4501	6701	N/A

UCVA: Uncorrected visual acuity, N/A: Not applicable, Mann-Whitney U test was used to compare UCVA in two phases. *P*<0.05 is statistically significant

Posterior capsule rupture occurred in seven eyes (1.7%) in the 2006 group and four eyes (2.5%) in the 2003 group. Retained suture was observed in eight eyes (2%) in the 2006 group and in three eyes (2%) in the 2003 group.

## DISCUSSION

In less than 5 years, cataract surgery techniques at Farabi Eye Hospital have changed. For example, the phacoemulsification rate of 25% in 2003 increased to more than 90%. Although “good” visual outcome (UCVA >6/18, WHO criteria) was achieved in both years, we showed that UCVA was better in 2006. During this period of transition, incorporation of new technology and adoption by surgeons of newer skills, patient satisfaction was maintained. Over the study period, the spectrum of patients broadened: patients from a broader age range and poorer systemic conditions were offered surgery and more myopic patients were scheduled for surgery (13.9% in 2003 vs. 19.3% in 2006) [Table 1]. At the same time, the volume of cataract surgery procedures increased. These data indicate the prevalent attitude of surgeons to increase the indications for surgery. Less than a decade ago, the standard approach for myopic patients was to postpone surgery in Iran, contrary to the recent approach, which is to offer the cataract-refractive surgery as soon as the least visually significant lens opacity is present. This exemplifies the role of health technology on the definition of health and the opportunity to improve quality of life.^7^,^8^

Gogate *et al*.^9^ and Ruit *et al*.^10^ compared phacoemulsification with manual SICS in two randomized controlled trials in India and Nepal, respectively, and reported that the techniques were comparable in efficacy and safety. Manual SICS has also been shown to cost less than phacoemulsification.^11^ Although in our center we had a sizable amount of SICS in 2003 (28.2%), this technique was not popularized in Iran. This could be attributed to opposition from ophthalmic leaders and popularization of phacoemulsification instead of SICS.

Although phacoemulsification is far more dependent on technology than the conventional procedures, overall cost-effectiveness and suitability of each technique vary based on location and facilities.^12^,^13^ For example, our study showed that the 2006 group mainly underwent surgery with local anesthesia (87.4%) on an outpatient basis (70.9%). This results in lower expenses due to less hospitalization, lower anesthesia-related charges and lower systemic complications attributable to general anesthesia.

The changes of surgical routines in our center are indicative of the process that is named “knowledge translation.” This dynamic process includes synthesis, dissemination, exchange and ethically sound application of knowledge to improve the health of citizens, to provide more effective health services and products and to strengthen the health care system.^14^ Knowledge transfer and exchange faces obstacles. Phacoemulsification was proposed by Kelman,^15^ but was popularized in the late 1980s. Leaming documented the transition in the technique of cataract extraction in the United States, in which there was an increasing preference for phacoemulsification, rising from 12% in 1985 to 86% in 1994.^16^,^17^ A number of surveys have examined the current practice and trends in cataract and refractive surgery worldwide, which show that the transition has taken place later in those countries.^18^–^21^ Yi *et al*.^18^ reported that there was an increasing trend in use of phacoemulsification in Australian cataract patients from 55% in 1994 to more than 90% in 1998. Oshika *et al*.^21^ reported that the rate of surgeons in Japan who prefer phacoemulsification increased from 59% in 1992, 90% in 1996 to 94% in 1999. For the developing countries, the same issues are true, and are more troublesome. The senior author (HH) recalls having to self-teach with an old phacoemulsification machine in the early 1990s.

Multiple models have been proposed to ease the process of knowledge transfer and exchange.^22^,^23^ A variety of reasons for lags in the acquisition of skills and technology from the West has been proposed: lack of attention to research studies in decision making, the effect of the international political atmosphere on technology transfer, presence of a bureaucratic system, costs and conflict of interest.^24^ It is noteworthy that some apparently unrelated issues such as reimbursement policy could act as a driving or hampering force in technology transfer. For example, if foldable IOLs were covered by the insurance system, it is expected that the phacoemulsification procedure would be popularized; otherwise, the surgeon might question the usefulness of phacoemulsification.

It is well known that becoming an advanced phacoemulsification surgeon is not easy. The learning curve for residents during their training experience for this procedure has been determined to be 80 cases.^25^ Our study documented a remarkable growth in terms of surgical skill attainment (competence acquisition): in a matter of 3 years, the rate of moderately and highly skilled phacoemulsification surgeons improved from less than 15% to over 75%. This could be attributed to key trainers, the educational nature of the setting and the thirst of the residents and fellows to perform phacoemulsification instead of conventional procedures. As a matter of fact, the leadership of the ophthalmology department changed in that time (2004–2005), and the attitude of the new chair (HH) was instrumental in popularizing phacoemulsification. Fear of stagnating due to lack of adoption of new technology and peer pressure have also been proposed as the possible driving forces in other settings.^26^ Academic centers and leaders are potentially indispensable to the knowledge transfer. Participation in congresses, sabbatical leave and inviting visiting professors to developing countries could amplify the knowledge transfer cycle.

The findings of this study should be interpreted carefully as it is possible that those who had undesirable visual outcome may have been more motivated to attend the follow-up visit. However, random selection for subject recruitment and comparability of the participants to non-participants in terms of basic factors indicates that our sample is representative of those who had surgery in 2003 and 2006.

In conclusion, we were able to objectively document the cataract surgery technique transition at Farabi Eye Hospital in the mid-2000s. This was accompanied by significant expansion of the spectrum of cataract surgery candidates and remarkable attainment of surgical skill. At the same time, the visual outcome improved and the patient satisfaction was maintained.
